# Aptamers as a Sensitive Tool to Detect Subtle Modifications in Therapeutic Proteins

**DOI:** 10.1371/journal.pone.0031948

**Published:** 2012-02-27

**Authors:** Ran Zichel, Wanida Chearwae, Gouri Shankar Pandey, Basil Golding, Zuben E. Sauna

**Affiliations:** 1 Laboratory of Hemostasis, Division of Hematology, Center for Biologics Evaluation and Research, Food and Drug Administration, Bethesda, Maryland, United States of America; 2 Laboratory of Plasma Derivatives, Division of Hematology, Center for Biologics Evaluation and Research, Food and Drug Administration, Bethesda, Maryland, United States of America; Beckman Research Institute of the City of Hope, United States of America

## Abstract

Therapeutic proteins are derived from complex expression/production systems, which can result in minor conformational changes due to preferential codon usage in different organisms, post-translational modifications, etc. Subtle conformational differences are often undetectable by bioanalytical methods but can sometimes profoundly impact the safety, efficacy and stability of products. Numerous bioanalytical methods exist to characterize the primary structure of proteins, post translational modifications; protein-substrate/protein/protein interactions and functional bioassays are available for most proteins that are developed as products. There are however few analytical techniques to detect changes in the tertiary structure of proteins suitable for use during drug development and quality control. For example, x-ray crystallography and NMR are impractical for routine use and do not capture the heterogeneity of the product. Conformation-sensitive antibodies can be used to map proteins. However the development of antibodies to represent sufficient epitopes can be challenging. Other limitations of antibodies include limited supply, high costs, heterogeneity and batch to batch variations in titer. Here we provide proof-of-principle that DNA aptamers to thrombin can be used as surrogate antibodies to characterize conformational changes. We show that aptamers can be used in assays using either an ELISA or a label-free platform to characterize different thrombin products. In addition we replicated a heat-treatment procedure that has previously been shown to not affect protein activity but can result in conformational changes that have serious adverse consequences. We demonstrate that a panel of aptamers (but not an antibody) can detect changes in the proteins even when specific activity is unaffected. Our results indicate a novel approach to monitor even small changes in the conformation of proteins which can be used in a routine drug-development and quality control setting. The technique can provide an early warning of structural changes during the manufacturing process that could have consequential outcomes downstream.

## Introduction

Therapeutic proteins now represent a significant segment of the pharmaceutical industry [1] and include some of the most innovative products which are on the cutting edge of clinical care. This class of therapeutics is clearly different from small molecule synthetic drugs. Protein-drugs are 100 to 1,000 times larger, have complex secondary and tertiary structures, and cannot be synthesized by chemical processes and have to be manufactured in living cells. Compared to small-molecule entities, the manufacture of biopharmaceuticals involves far larger numbers of batch records (>250 vs. <10), product quality tests (>2,000 vs. <100), critical process steps (>5,000 vs. <100), and process data entries (>60,000 vs. <4,000) [2]. Analytical testing is an indispensible part of the pre-clinical development as well as the routine manufacture of any pharmaceutical and recent trends make such testing even more critical. The globalization of the industry means that the different steps in the manufacture of a single product occur at several locations and even in different countries. This poses challenges in quality control and has seen the emergence (and adoption) of approaches like quality by design [3], which rely heavily on exhaustive analytical testing. The lack of analytical techniques to comprehensively characterize large molecule biotherapeutics also lay at the heart of the debate on whether or not to permit the development and licensure of biosimilars [4]. Legislative authorities in Europe [5] as well as the US [6] have now ratified pathways for the approval of biosimilars. An examination of the EMA experience [7] shows that the paradigm used for small molecule generics cannot be used for biosimilars. The classical generic approach worked well for chemically derived products because characterization by analytical methods was determined to be a good predictor of the biological and clinical properties of the drug. The lack of suitable techniques to effectively compare the biosimilar with the reference product necessitates more extensive clinical trials than would otherwise be warranted [8].

Table 1 lists the techniques used to characterize protein therapeutics. A number of technologies can be used to characterize lower levels of protein organization (such as the primary and secondary structures) with a high degree of accuracy and sensitivity. Significant improvements in mass spectrometry over the last decade allow the determination of variations in post-translational modifications. Similarly the biophysicist's toolkit offers a choice of technologies to quantify protein-protein and protein-substrate interactions. Moreover robust assays to measure the biochemical activity of most protein products have been well established. A significant gap, however, remains in monitoring the tertiary and quaternary structures of proteins during drug development and manufacture [9]. Techniques currently used to determine the structures of proteins such as X-ray crystallography and NMR do not lend themselves to routine use during the manufacturing process. Issues of cost, time and technical skills aside these techniques fail to capture the heterogeneity of the product, which is a hallmark of biotherapeutics. It has, for example, been estimated that as many as 10^8^ possible product-variants and impurities for a monoclonal antibody product can occur [2].

**Table 1:** Analytical techniques used to characterize protein therapeutics.

Composition, primarystructure	Peptide mapping (mass spectroscopy), peptide mass fingerprint (mass spectroscopy), amino acid sequencing
Higher-order structure	Spectroscopy, thermal stability testing
Conformation	ELISA, epitope mapping
Post-translational modifications (e.g., glycosylation)	Mass spectroscopy, ion-exchangechromatography
Size, detection of aggregates	Gel electrophoresis, size-exclusion chromatography, analytical ultracentrifugation
Binding	Cell assays, spectroscopy, SPR, ELISA
Biological activity	Cell assays, animal models

Epitope mapping using antibodies is one of the few methods currently available to monitor the conformation of a protein product [10]–[12]. In recent years synthetic nucleic acid reagents, called aptamers have emerged as surrogates to antibodies and appear to be particularly suited for bioanalytical applications [13]. Aptamers are engineered through repeated rounds of *in vitro* selection, SELEX (systematic evolution of ligands by exponential enrichment) to bind virtually any molecular target such as small molecules, proteins, nucleic acids, and even cells, tissues and organisms. The *in vitro* selection process confers significant advantages to aptamers over antibodies. The three topical thrombins currently marketed in the US were not developed as biosimilars, nonetheless these products are used for the same indications and are generally regarded as interchangeable in a clinical setting [14]. However, these products are derived from different sources and have different manufacturing processes [15] and offer a good real life model system to evaluate the panel of aptamers for discriminating between different potential conformations of similar proteins. Here we present proof-of-principle that aptamers can be used to characterize conformational epitopes on therapeutic proteins. We have used six anti-human thrombin aptamers to characterize epitopes on three thrombin products currently marketed. The aptamers clearly distinguish between the human and bovine thrombin products. However the two human thrombin products (one purified from human plasma and the other manufactured by recombinant DNA technology) show comparable binding to all six aptamers. We also heat treated the human thrombin products, using a procedure similar to that resulting in enhanced immunogenicity in Factor VIII (FVIII) [16], [17]. This treatment did not affect thrombin activity or the binding to an anti-thrombin antibody. The panel of anti-thrombin aptamers however detected significant alterations in the conformation of thrombin epitopes.

## Results

### Using aptamers as surrogate antibodies in an ELISA assay

**Figure 1:**
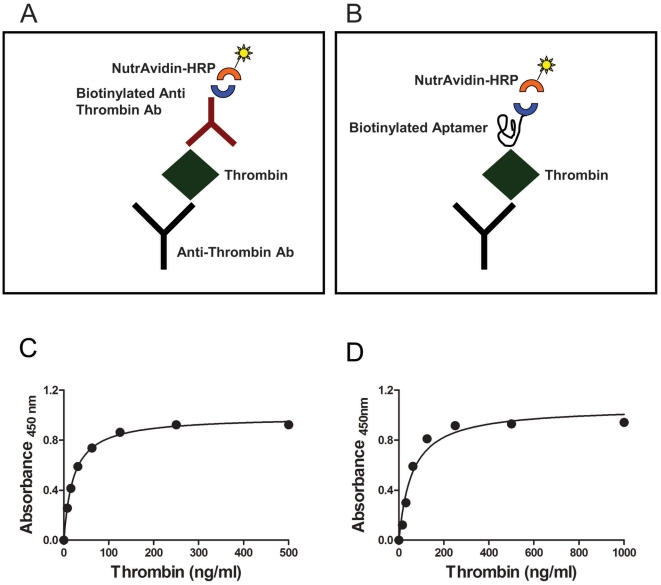
ELISA based binding assays using aptamers as surrogate antibodies. (***A & B***) Illustrations showing assay platforms using either polyclonal antibody (***A***) or aptamers (***B***) to detect thrombin. Both assays use an identical anti-thrombin monolonal antibody as the capture antibody and identical reagents for detection (NutrAvidin-HRP and TMB). Assays used in Fig. 6 however use a polyclonal antibody as the capture antibody, one of four monoclonal antibodies as detectors and anti-mouse-HRP as a secondary antibody (***C***) ELISA assay showing the limit of detection for purified human thrombin using the antibody based assay illustrated in (***A***). (***D***) ELISA assay showing the limit of detection for purified human thrombin using the aptamer based assay illustrated in (***B***).

**Figure 2:**
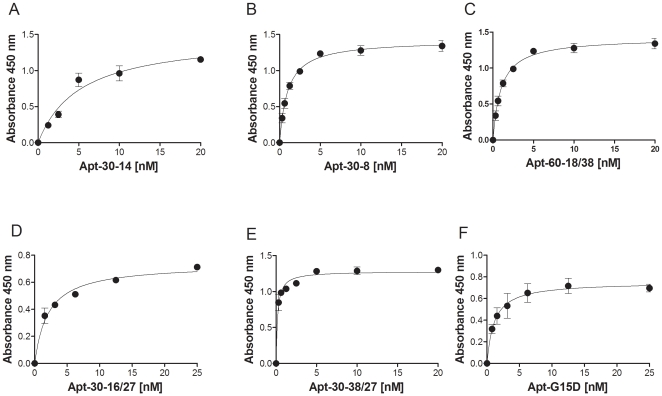
Affinities of the six aptamers used in this study determined using the aptamer based ELISA. A fixed concentration of thrombin (Evithrom), 150 nM was captured by the anti-thrombin monoclonal antibody, 5 µg/ml. Binding of increasing concentrations of the six anti-thrombin aptamers (***A***, Aptamer 30-14, ***B***, Aptamer 30-8, ***C***, Aptamer 60-18/38, ***D***, Aptamer 30-16/27, ***E***, Aptamer 30-38/27 and ***F***, Aptamer G15D) was determined using the assay depicted in Fig. 1B. Data presented shows the mean (± SD) of three experiments where the aptamers were generated independently for each experiment.

We identified six anti-thrombin DNA aptamers previously described to bind different thrombin epitopes with varying affinities [18], [19]. The affinities of these anti-thrombin aptamers for thrombin have been determined using different assays and there have been some discrepancies (see [18]–[20]). For consistency of results it is very important that the conditions for the generation of aptamers be rigorously adhered to. Moreover the assay we developed uses biotinylated aptamers, which could affect the affinity. The primary sequences of the six polynucleotides used in this study are depicted in Table 2 and the conditions for generating the aptamers are given in the Materials and Methods. The aptamers were first evaluated using a sandwich ELISA. This assay was standardized using an anti-thrombin monoclonal capture antibody and a biotinylated polyclonal anti-thrombin detector antibody (Fig. 1A). In modifying this assay, we used the biotinylated anti-thrombin aptamers in lieu of the detector antibody (Fig. 1B). Both assays use the same monoclonal antibody for capture and identical reagents, NutraAvidin-HRP and TMB. Using recombinant thrombin expressed in CHO cells (Recothrom), we demonstrate that the limit of detection for thrombin is approximately 10 ng/ml using either the antibody or the aptamer (Figs. 1 C&D). We therefore used a fixed concentration of the thrombin (5 µg/ml) to determine the relative affinities of the six aptamers used in this study (Fig. 2 and Table 3). The assay is robust, reproducible and the six aptamers bind to the thrombin with apparent K_d_ values ranging from 0.58±0.01 nM to 2.75±0.36 nM (Table 3). Prior to each experiment the oligonucleotides were denatured and then allowed to refold under controlled conditions (see Materials and Methods for details) to form the aptamers. The SD thus represents not only the variation in the assay *per se* but also the variability in process of generating the aptamers.

**Table 2:** Sequences of aptamers used in this study.

Aptamer	Sequence
30-14	5′AGATGCCTGTCGAGCATGCTGTTGTGGTAGGGTTAGGGATGGTAGCGGTTGTAGCTAAACTGCTTTGTCGACGG′3
30-16/27	5′TACCGTGGTAGGGAAGGTTGGAGTGTA′3
30-38/27	5′ACCCGTGGTAGGGTAGGATGGGGTGGT′3
60-18/38	5′CAGTCCGTGGTAGGGCAGGTTGGGGTGACTTCGTGGAA′3
G15D	5′GGTTGGTGTGGTTGG′3
30-8	5′AGATGCCTGTCGAGCATGCTGTGAATAGGTAGGGTCGGATGGGCTACGGTGTAGCTAAACTGCTTTGTCGACGGG′3

**Table 3:** Kinetic parameters for the binding of aptamers to Recothrom.

Aptamer	V_max_	Apparent K_d_
60-18/38	1.58±0.01	1.48±0.05
30-14	1.38±0.05	2.75±0.36
30-8	1.29±0.01	0.58±0.01
30-16/27	0.819±0.0196	1.54±0.42
30-38/27	0.18±0.03	1.28±0.03
G15D	0.68±0.02	1.48±0.15

### Comparing the binding of aptamers to thrombin products

**Figure 3:**
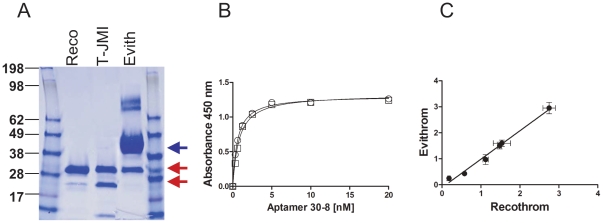
Comparing three topical thrombin products using binding assays with aptamers as detector reagents in an ELISA format. (***A***) The three topical thrombin products were electrophoresed on a 12% NuPAGE gel and stained using Colloidal Blue®. The thrombin and HSA (used as an exipient) bands are indicated with red and blue arrows respectively. (***B***) Kinetics of binding of the Aptamer 30-8 to the purified human thrombin products Recothrom (squares) and Evithrom (circles). (***C***) The affinities (apparent K_d_s) of all six aptamers to both Recothrom and Evithrom were determined as shown in (***B***). The apparent K_d_ values for Recothrom (X-axis) were plotted against those for Evithrom (Y-axis) and were shown to be highly correlated (r = 0.996, *P*<0.05).

There are three topical thrombin products in the market. Prior to 2007 the only topical thrombin available was one derived from bovine plasma (Thrombin JMI), since then a human-plasma derived thrombin (Evithrom) and a human recombinant thrombin (Recothrom) have been approved [14]. Bovine thrombin is produced by extracting prothrombin from bovine plasma. After activation to thrombin, the product undergoes a chromatographic purification process that includes ion exchange and viral filtration [21]. Human thrombin is derived from human plasma [22] while human recombinant thrombin is produced via recombinant DNA technology from genetically modified chinese hamster ovary (CHO) cells [23]. Studies have indicated that the efficacies of the human thrombins are comparable to that of the bovine thrombin which has been used in the clinic for decades [24], [25]. Fig. 3A shows a colloidal blue stained gel with the three thrombin products. All three products are highly purified, however the major protein component of the plasma derived human product is human serum albumin (HSA) which is used as a stabilizing agent [22]. No proteins are added to the bovine thrombin or the human recombinant thrombin drug-products [21], [23]. Despite these differences in manufacturing processes and excipients, the binding of the Aptamer 30-8 to the two human thrombin products is almost indistinguishable (Fig. 3B). Furthermore when we compared the kinetics of the binding of all six aptamers to the two human thrombin products we found an extremely high correlation (r = 0.996, *P*<0.05) (Fig. 3C). Interestingly none of the six aptamers binds to the bovine thrombin (data not given).

### Aptamers can detect subtle conformational changes in human thrombin products

**Figure 4:**
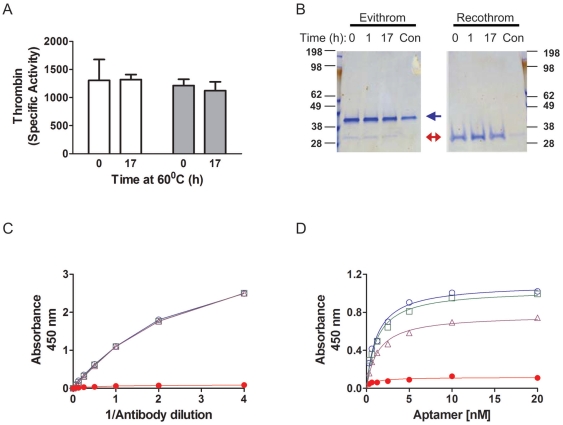
Conformational changes in thrombin following heat treatment in the presence of 60% sucrose. The two purified human topical thrombin products were heated for up to 17 h at 60°C in the presence of 60% sucrose in duplicate. (***A***) The catalytic activity of the Recothrom (clear bars) and Evithrom (grey, filled bars) samples was determined at time 0 and 17 h; the mean activity (± SD) of three independently treated samples is depicted. (***B***) Following heat treatment both samples were electrophoresed on a 12% NuPAGE gel and stained using Colloidal Blue®. As a control (Con) the thrombin samples were heated at 60°C in PBS the absence of sucrose. (***C***) The binding kinetics of the heat-treated thrombin at time 0 h (Blue open circles) and 17 h (purple open triangles) were determined using the antibody based ELISA illustrated in Fig. 1A. Samples heated in the presence of PBS alone are shown as red filled circles. (***D***) The binding kinetics of the heat-treated thrombin at time 0 h (Blue open circles), 1 h (green open squares) and 17 h (purple open triangles) were determined using the aptamer based ELISA illustrated in Fig. 1B. Samples heated in the presence of PBS alone are shown as red filled circles.

**Figure 5:**
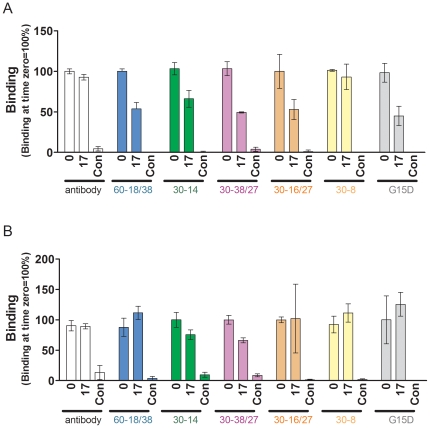
Heat treatment of human thrombins in the absence and presence of 60% sucrose. The two purified human topical thrombin products were heat-treated as described in Fig. 3. Control samples were heated in for 17 h at 60°C in PBS (in the absence of 60% sucrose). Binding of either the antibody or each of the six aptamers was monitored to (***A***) Recothrom or (***B***) Evithrom at time 0 and 17 h. The percent of the binding at time 0 (mean ± SD, n = 3) is represented. The aptamers used are shown on the figure.

There are many instances where a small change in manufacturing process significantly impacts a protein therapeutic (see [2] for numerous examples). For example, a manufacturing change involving heat treatment of FVIII resulted in a significant increase in the frequency of inhibitors even though other product parameters (such as specific activity) remained unaffected [26]. It was subsequently determined that the heat treatment likely resulted in subtle alterations in the conformation leading to the exposure of some epitopes [17]. We therefore investigated whether a comparable treatment of the human thrombins would affect the binding of the panel of aptamers used in this study. Evithrom and Recothrom were incubated at 60°C for a maximum of 17 h in the presence of 60% sucrose buffer as has been described previously (see Materials and Methods and [16] for details). Consistent with previous studies [16] we found that heating in the presence of 60% sucrose afforded protection and there was no significant effect on the specific activity of either product (Fig. 4A). On the other hand incubation at 60°C in PBS alone resulted in both protein degradation (Fig. 4B) as well as complete abrogation of thrombin activity (data not given). Similarly the antibody based binding assay (see Fig. 1A for assay format) showed no detectable difference in the binding of the polyclonal antibody, to thrombin following up to 17 h of incubation at 60°C in the presence of 60% sucrose buffer (Fig. 4C). On the other hand the positive control (heat treatment in the presence of PBS alone) results in antibody binding being almost complete abolished. The aptamer based assay shows time-dependent changes in the binding kinetics when the thrombin is incubated in the presence of sucrose (Fig. 4D). We compared the effect of heat treatment of both the plasma derived and recombinant thrombin on the binding of all six aptamers (Fig. 5). The binding of five out of six aptamers showed a statistically significant decrease in binding compared to untreated recombinant thrombin. However, the binding of only two aptamers was affected when the plasma derived thrombin preparation was heat treated.

**Figure 6:**
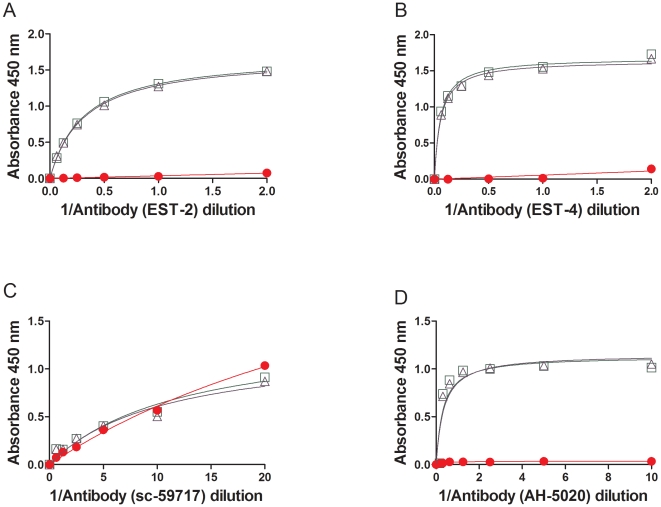
Effect of heat treatment of thrombin in the presence of 60% sucrose monitored using anti-thrombin monoclonal antibodies. The binding kinetics of the heat-treated thrombin (Recothrom) at time 0 h (Green open squares) and 17 h (purple open triangles) were determined using an antibody based ELISA. The polyclonal anti-thrombin antibody, PAHT-S-B was used as the capture antibody and a panel of monoclonal antibodies as detector antibodies as described in the Materials & Methods. The binding kinetics of the mouse anti-thrombin antibodies EST-2 (***A***), EST-4 (***B***), sc-59717 (***C***) and AH-5020 (***D***) are depicted.

We evaluated four commercially available anti-thrombin monoclonal antibodies for their capacity to distinguish between control and heat-treated thrombin (Fig. 6). In the experiment depicted in Fig. 3B a monoclonal antibody was used to capture thrombin and a polyclonal antibody used as the detector. In the ELISA assays shown in Fig. 6, a sheep anti-human thrombin polyclonal was used to capture the thrombin and a panel of mouse anti-thrombin monoclonal antibodies was used to detect the protein. The monoclonal antibody EST-2 recognizes thrombin alone as well as the thrombin-antithrombin complex [27]. The EST-4 on the other hand binds thrombin but not the thrombin-antithrombin complex and it has been suggested that this monoclonal antibody recognizes an epitope near the active site [27]. Both these antibodies were obtained by the fusion of α-thrombin immunized spleen cells with a myeloma cell line. We also used two additional mouse anti-thrombin antibodies (sc-59717 and AH-5020) [28], [29] raised against purified full-length native human thrombin in these assays (Fig. 6C&D). We selected monoclonal antibodies that were raised using protein molecules rather than peptides as these are more likely to be conformation sensitive. However all the four monoclonal antibodies showed comparable binding kinetics for the control and heat treated thrombin. In fact the monoclonal antibody sc-59717 continued to bind denatured thrombin (heated at 60°C for 17 h in PBS).

### Using aptamers in label free characterization of the human thrombin products

**Figure 7:**
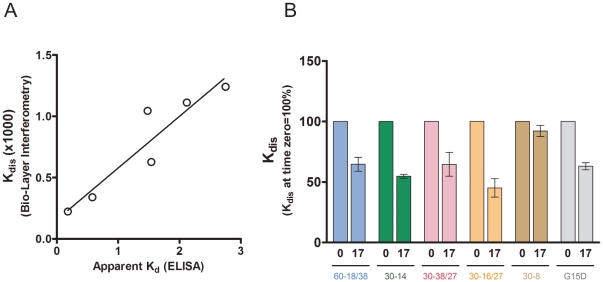
Estimating the binding of aptamers to thrombin in a label free assay. The binding of aptamers to Recothrom was monitored in real time using BLI and the kinetic parameters obtained using the Octet data analysis software version 7.0. (***A***) Correlation between K_dis_ values obtained in the label-free assay with the apparent K_d_s estimated using the ELISA based assay (r = 0.842, *P*<0.05). (***B***) Effect of heat-treatment of human thrombins on the k_dis_ for all 6 aptamers. Recothrom was heat-treated as described in Fig. 3 and the k_dis_ determined following heat treatment for 0 and 17 h. The percent of the binding at time 0 is represented. Data presented is the mean (± SD) of three independent experiments.

ELISA based assays are simple, and do not require much specialized equipment or skills and have found wide application in research, clinical laboratories as well as during drug development. These assays however require capture and detection antibodies, are subject to wash steps, multiple incubations, and serial dilutions for each sample; factors that affect precision, accuracy and turn around time. Moreover, in recent years label free technologies have been found to be advantageous over methods where the kinetic constants are derived indirectly and are assay dependent [30]. Several technologies are available that permit the label-free determination of kinetic constants including the association rate constant (k_on_), dissociation rate constant (k_dis_), and equilibrium dissociation constant (KD). Here we utilized BioLayer Interferometry, to measure in real-time the aptamer-thrombin interactions using streptavidin. In this assay, the biotinylated aptamer is bound to the biosensor and the rate of binding to thrombin was found to be concentration dependent as has been shown previously for protein-protein interactions such as those between an antibody and its ligand [30]. We used this data for base-line correction and to obtain the k_on_ and k_dis_ and KD values using the Octet Software (data not given). Using the recombinant human thrombin, Recothrom as an example we found that the K_dis_ values obtained in the label-free assay correlate (r = 0.842, *P*<0.05) with the apparent K_d_s estimated using the ELISA based assay (Fig. 7A). A similar correlation was obtained when we compared the KD values from the label free assay with the K_d_ values from the ELISA based assay (data not given). The advantage of using the k_dis_ values is that while the apparent K_d_ and KD determinations are concentration dependent and require accurate estimations of protein and nucleotide the k_dis_ is independent of the concentration of the ligand. This can be useful as the concentration of samples can change during processing. The heat treatment described in the previous section involves dilution and incubation in the 60% sucrose medium. We therefore compared the change in the dissociation rate constants (k_dis_) for the six aptamers following heat treatment of Recothrom (Fig. 7B). A statistically significant change in the k_dis_ is observed in 4 of the 6 aptamers as a consequence of heat treatment, consistent with our results using the ELISA based assay (Fig. 5).

## Discussion

In this study we characterize aptamers as surrogate antibodies for the identification of changes in the conformation of a protein during the manufacturing process and during storage. This is important because the inability to detect changes in the conformation of a protein-product can have consequences even if the activity is unaffected. For example even subtle changes in conformation can result in new epitopes on the protein, leading to immunogenicity [31]. Immunogenicity is currently a significant impediment to the successful development of therapeutic proteins, as the development of inhibitory antibodies can reduce the efficacy and sometimes even result in life threatening safety issues [32]. Similarly, comparison of biosimilars to reference standards using bioanalytical techniques is a key component of the licensure of such products [5]. Here we have used a panel of aptamers to compare three different thrombin products vis-à-vis the conformational epitopes.

The affinity of a panel of six anti-thrombin aptamers for the protein was determined using an ELISA like assay which is illustrated in Fig. 1B. A single monoclonal antibody was used to capture the thrombin molecules which were then detected using either a polyclonal anti-thrombin antibody or one of the six anti-thrombin aptamers. In another set of experiments, the anti-thrombin polyclonal antibody was used to capture the thrombin molecules and the binding kinetics of four anti-thrombin monoclonal antibodies were evaluated. By standardizing the assay we were able to obtain excellent signal to noise ratio for all six aptamers, comparable to that for the antibody based assay. Moreover the limit of detection for thrombin is also comparable regardless of whether an aptamer or antibody was used and we obtained consistent results for binding kinetics where the per-cent CV ranged from 9 to 14% with an average of 12.4% (Table 3).

Both the polyclonal detector antibody and all six aptamers bound the Evithrom with high affinity (Fig. 2 & 3B) but showed almost no binding to Thrombin JMI. Both these thrombin products are purified from plasma but the former is of human origin and the latter of bovine origin. This demonstrates significant specificity as the bovine and human thrombins are 89% homologous and show 81% sequence identity [33]. Moreover in bioassays as well as clinical studies the bovine and human thrombins have been shown to exhibit comparable activity. A comparison of the binding affinities of the aptamers for Evithrom and Recothrom (Fig. 3C) showed a very high correlation (r = 0.996, *P*<0.05). Evithrom and Recothrom are both human thrombin products, however while the former is purified from plasma the latter is a recombinant protein expressed in CHO cells [22], [23]. This result would suggest that these two products while obtained by two very different processes have remarkably similar conformations given that the aptamers are extremely sensitive to subtle changes (Figs. 4D, 5 & 7B).

We have alluded to a case study from the 1990s where an additional step of pasteurization was used to inactivate non-lipid-enveloped viruses in the manufacture of FVIII led to the development of inhibitory antibodies at a very high frequency leading to the withdrawal of the product from the market [26]. Subsequent studies have suggested that the change in the manufacturing process exposed sites in the C2 domain of the FVIII leading to immunogenicity [17]. This case vividly illustrates the significance of the need for techniques that can routinely detect subtle changes in conformation because not all changes affect product activity in *in vitro* assays. When we used a similar heat-treatment for the two human thrombin products we found no significant change in activity even after 17 h (Fig. 4A). Nor could assays using the antibody-based ELISA platform detect any changes in the affinity of the anti-thrombin antibody (Figs. 4C & 6) following heat-treatment when the protein is protected with 60% sucrose. As a control we demonstrate that heat-treatment in the absence of sucrose completely abolishes antibody binding (Fig. 4C & 6) in all but one of the antibodies screened. Aptamers on the other hand show small but significant differences in binding during heat-treatment of Recothrom in the presence of 60% sucrose and these changes are time dependent (Fig. 4D). It is interesting to note (a) that the magnitude of change varies for different aptamers and (b) the binding of only some aptamers is affected (Fig. 5A). This suggests that the aptamers recognize different epitopes or conformations on the protein. We obtained similar results in a label-free platform using BioLayer Interferometry (Fig. 7B). This panel of aptamers also showed alterations in binding to Evithrom following heat treatment, except that the binding of fewer aptamers is affected (Fig. 5B). It is noteworthy that all the aptamers affected by the heat-treatment of Evithrom are also affected in Recothrom. It is plausible that the high concentration of HSA in the Evithrom provides additional protection to the protein during heat treatment. Taken together these results suggest that a suitable panel of aptamers can be used to detect small changes in protein conformation not detectable by other methods.

We used a panel of six aptamers in this study; the nature, number and choice of antibodies/aptamers is likely to be an important factor in the success of epitope mapping for detecting subtle changes in the conformation of individual protein products. Depending on the complexity of a therapeutic protein the panel of aptamers used to map a protein can readily be expanded, the same is not true of antibodies. In addition protein-specific aptamers are selected from a very large pool of conformations [34]–[36]. Consequently a larger repertoire of aptamers representing more epitopes can be generated for a target protein. Aptamers are also likely to be more useful in a quality control environment [37]–[41]. In addition, we determined in this study that the presence of excipients (including proteins other than the active ingredient) has no significant effect on the binding of aptamers to the thrombins. This observation could potentially be of consequence in comparing biosimilars to their reference standards. The primary hurdle for the developer of any biosimilar is that there is normally no direct access to originator companies' proprietary data. Thus, the developer of a biosimilar has to retrieve the reference protein as a finished drug product, purify the drug substance and reverse engineer the process [42]. This in turn has raised concerns that the reverse engineered ‘reference standard’ could itself be altered from the original active ingredient manufactured by the innovator. As aptamer binding is unaffected by the excipients, the assays described here provide a means of comparing the active ingredient in the finished drug product with the reverse engineered reference standard.

This study demonstrates that aptamers can be used to characterize changes in the tertiary structure of proteins. This has potential applications as an analytical test during the development of therapeutic proteins and to monitor the effect of manufacturing changes and storage. A range of biochemical and biophysical tests are available to characterize protein-drugs, however analytical methods to rapidly and routinely monitor subtle changes in the tertiary structure represent a critical lacuna. The need for such methods is particularly acute in the development of biosimilars. Here we have provided proof-of-principle that aptamers are stable reagents that can be used to design sensitive and robust analytical tests to detect small changes in the conformation of therapeutic proteins.

## Materials and Methods

### Materials

Recombinant human thrombin was from Zymogenetics (Seattle, WA), and plasma-derived human thrombin was from Omrix (Israel). Aptamers were synthesized by the standard phosphoramidite method on an ABI model 394 oligonucleotide synthesizer (Facility for Biological Research, CBER, FDA), purified by reversed phase HPLC and lyophilized. Aptamers were used either unmodified or in the biotynilated forms as indicated in the text. Table 2 lists the aptamers used in the study.

### Antibody based binding assay

ELISA plates (MAxisorp, Nunc) were coated with 50 µl mouse anti-human thrombin monoclonal antibody (Hemtech AHT-5020) diluted to 3.9 µg/ml in coating buffer (50 mM Na_2_CO_3_, pH 9.6), and incubated overnight at 4°C. Plates were washed in PBST and blocked for 1 hr at 37°C with 300 µl per well of 2% (W/V) BSA in Tris-NaCl pH 7.6 (TSTA). After washing, plates were incubated with 5 µg/ml of the indicated thrombin preparation diluted in PBST (50 µl, in duplicate) for 1 hr at 37°C. Plates were washed 4 times with PBST and incubated with biotinylated sheep anti-human thrombin polyclonal antibody (50 µl, Hemtech PAHT-S-B) diluted 1∶4000 in PBST for 1 hr at 37°C. After additional washing with PBST, the plates were incubated with 50 µl of HRP-Nutravidin (Pierce) diluted 1∶10000 in PBST for 1 hr at 37°C. Finally, plates were washed 4 times with PBST, and the chromogenic reaction was started using 100 µl 3,3′,5,5′-tetramethylbenzidine (TMB). Reaction was stopped with 100 µl 0.25 M H_2_SO_4_ and absorbance was measured at 450 nm. To determine the binding kinetics of a panel of anti-thrombin monoclonal antibodies, the ELISA plates (MAxisorp, Nunc) were coated with 50 µl sheep anti-human thrombin polyclonal antibody (Hemtech PAHT-S-B) diluted to 10 µg/ml in coating buffer (50 mM Na_2_CO_3_, pH 9.6), and incubated overnight at 4°C. Plates were processed as described above and incubated with 5 µg/ml of the indicated thrombin preparation diluted in PBST (50 µl, in duplicate) for 1 hr at 37°C. Plates were then washed 4 times with PBST and incubated for 1 hr at 37°C with serial dilutions of one of four mouse anti-human thrombin monoclonal antibodies (EST-2, EST-4 both from American Diagnostica Inc; sc-59717, Santa Cruz Biotechnology, Inc. or AHT-5020, Haematologic Technologies, Inc). After additional washing with PBST, the plates were incubated with 50 µl of HRP-conjugated anti-mouse antibody (Rockland) diluted 1∶10000 in PBST for 1 hr at 37°C. Finally, plates were washed 4 times with PBST, and the chromogenic reaction was started using 100 µl TMB. Reaction was stopped with 100 µl 0.25 M H_2_SO_4_ and absorbance was measured at 450 nm.

### Aptamer based binding Assay

ELISA plates (MAxisorp, Nunc) were coated with 50 µl mouse anti-human thrombin monoclonal antibody (Hemtech AHT-5020) diluted to 3.9 µg/ml in coating buffer (50 mM Na_2_CO_3_, pH 9.6), and incubated overnight at 4°C. Plates were washed in PBST and blocked for 1 hr at 37°C with 300 µl per well of 2% (W/V) BSA in Tris-NaCl pH 7.6 (TSTA). After washing, plates were incubated with 5 µg/ml of the indicated thrombin preparation diluted in PBST (50 µl, in duplicate) for 1 hr at 37°C. Biotinylated aptamers were heated to 95°C for 10 minutes and allowed to refold at room temperature (RT) for another 20 minutes. Folded aptamers serially diluted in folding buffer (25 mM Tris, 10 mM NaCl, 1 mM MgCl_2_ pH = 7.4) were loaded to the plate and incubated at RT protected from light for 1 hr. The plate was washed 4 times with aptamer folding buffer containing 0.005% Tween-20. The plates were incubated with 50 µl of HRP-Nutravidin (Pierce) diluted 1∶10000 in dilution buffer for 1 hr at RT. After a final wash the chromogenic reaction was started using 100 µl TMB. Reaction was stopped with 100 µl 0.25 M H_2_SO_4_ and absorbance was measured at 450 nm.

### Stability Assay

Recombinant and plasma derived thrombin were diluted to 30 µg/ml in sucrose buffer (60% w/v sucrose, 0.1 M glycine, pH = 7.3). Control samples were diluted accordingly in PBS. Samples (1.4 ml) were incubated at 60°C for 0, 1 and 17 hr and maintained at −80°C until analyzed.

### Activity assay

Two-fold dilution of calibrator thrombin (25 µl, Thrombinoscope, Netherlands) in the range of 1000-0.1 nM or unknown samples were added to 96-well plates pre-loaded with 25 µl Z-GGR-AMC substrate at 800 mM in reaction buffer (20 mM Hepes, 150 mM NaCl, 0.1% BSA, pH7.4). The increase in fluorescence intensity, which is proportional to thrombin activity, was monitored continuously at 37°C by automatic reading every 30 sec up to 30 min using fluorescence reader (Tecan) with an excitation wavelength of 380 nm and an emission wavelength of 430 nm. Thrombin concentrations were expressed as the rate development of fluorescence intensity [arbitrary fluorescence units (FU)], calculated for every reading (FU min^−1^).

### Determining the Binding Affinity of aptamers using Bio-Layer Interferometry

Bio-Layer Interferometry (BLI), a label-free technology, was used for measuring the binding of aptamers with thrombin samples. The affinity measurements were performed with ForteBio Octet RED96 equipped with streptavidin (SA) biosensor tips (ForteBio, Inc., Menlo Park, CA, USA). The assays were maintained at a temperature of and the speed of 30°C and 1000 rpm respectively. Streptavidin-coated biosensor tips were pre-wet for 15 min. Then the tips were loaded with 250 nM–500 nM of biotinylated aptamer for 5 min. The typical resulting capture levels of eight biosensors were in the range of 0.4–0.5 nm. The association (k_on_) and dissociation (k_dis_) were then established by dipping the biosensors for 10 mins in various concentrations of thrombin samples dispensed in 96-microwell plates (Fisher Scientific, Turnburry Drive, Hanover Park, IL, USA) at a volume of 200 µl per well. Data were processed and analysed using the Octet data analysis software version 7.0 (ForteBio). The binding profile of each aptamer was shown as “nm” shift. This shift is a comparison of the shift in the interference patterns of light reflected from a reference layer within the biosensor versus molecules as the bind to the biosensor tip. The results were summarized as KD which was calculated from “KD = k_on_/k_dis_”, where k_on_ is the ‘on rate’ or association and k_dis_ is the ‘off rate’ or dissociation.

### Statistical analysis

Stability experiment was conducted in three independent repeats. Statistical analysis was carried out using repeated analysis of variance (ANOVA) performed with GraphPad InStat 3 software. Differences were considered significant when *P* was <0.05.
